# Physicochemical and Functional Properties of Snack Bars Enriched with Tilapia (*Oreochromis niloticus*) By-Product Powders

**DOI:** 10.3390/foods10081908

**Published:** 2021-08-17

**Authors:** Yasinta Zulaikha, Shuai-Huei Yao, Yu-Wei Chang

**Affiliations:** 1 Department of Food Science, National Taiwan Ocean University, Keelung City 20224, Taiwan; yasintazulaikha@gmail.com (Y.Z.); 00639034@email.ntou.edu.tw (S.-H.Y.); 2 Institute of Food Safety and Risk Management, National Taiwan Ocean University, Keelung City 20224, Taiwan

**Keywords:** snack bars, tilapia by-products, antioxidant, ACE inhibitor, antibacterial

## Abstract

This research aimed to evaluate tilapia by-product powders as a novel food ingredient and the suitable cooking method for snack bar (SBs) production. Tilapia by-product powders were made by two processing methods; one powder was oven-dried as tilapia dry powder (TDP) and another was bromelain-hydrolyzed and then freeze-dried as tilapia hydrolysate powder (THP). SBs were prepared by incorporating tilapia dry powders (TDP or THP; 10%). SBs were further separated in two different cooking methods, namely unbaked and baked ones. The baked SBs had yellow and darker coloration (L* value ranged from 66.38 to 76.12) and more reddish color (a* value range from −1.26 to 1.06). Addition of tilapia by-product powders significantly (*p* < 0.05) increased the protein content of the original SB from 21.58 to 32.08% (SB + THP). Regarding DPPH scavenging activity, the control group showed the lowest activity, followed by SB + TDP and SB + THP with the highest activity (*p* < 0.05), with DPPH scavenging activity ranged from 12.40 to 26.04%. The baking process significantly (*p* < 0.05) increased the angiotensin converting enzyme (ACE) inhibitory activity of the SBs. In particular, the SB + THP group showed the highest activity (17.78%). All samples exhibited antibacterial activity against *Staphylococcus aureus*, and the SB + THP group showed the highest activity (15.08 ± 1.95 mm growth inhibition). Based on principal component analysis, four principal components (nutraceutical pigmentation, physical characteristics, nutrition value, and greater dehydration) were contributed towards the physicochemical and functional properties of the SBs. The overall results suggested that tilapia by-product powders can be potential ingredients for adding functional values to food products.

## 1. Introduction

Snack bars (SBs), well-known as cereal bars, have been commonly consumed worldwide because they provide instant energy and are convenient to carry around. SBs moderate direct hunger and influence people’s nutritional status, which is commercially and nutritionally interesting [1]. Most consumers care for their diet and health. Thus, eating SBs can be a source of intake of beneficial nutrients, such as fiber, protein, minerals, and vitamins [2].

SBs are common oat (*Avena sativa*)-based products, a cereal technological feature that also supplies health benefits and cholesterol-lowering properties associated with β-glucan, a soluble-type dietary fiber [3]. However, oat-based SBs are typically deficient and limited in their amino acid profile, especially in threonine and methionine. This condition can be improved by adding complementary protein sources such as legume or animal proteins (good sources of threonine and methionine), increasing the protein and fiber content and improving the bioactive content in the product [4].

Animal and plant proteins have different effects on muscle health. In addition, dietary proteins from different food sources are usually different in their protein content, amino acid composition, and protein digestibility. Animal-based foods are the primary source of high-quality protein. Previously, studies have shown that higher animal protein intake is associated with greater muscle mass and less muscle loss in older Americans and Europeans [5].

Functional compounds in animal and plant-based food products and by-product supplementation have been developed by food industries [6]. One of the cases is in Nile tilapia (*Oreochromis niloticus*) industries. Tilapia is one of the main freshwater fish species that have a significant contribution to global aquaculture growth. In the tilapia fillet industry, the fillet yield is approximately 30%, whereas the other parts of tilapia, tilapia by-products, are identified as waste or under-utilized biomaterials. Waste management could be a strategy to reduce food waste’s economic, social, and environmental impacts. It can reduce food wastage, redistribute unsold or excess food, and recycle/treat food waste/by-products [7].

Tilapia dry powder (TDP) from the frame with meat fit to bones is relatively easy to prepare. This powder is not only affordable but also containing high-quality nutrients, incredibly high levels of essential amino acids (histidine, lysine, threonine, methionine, valine, and leucine) and polyunsaturated fatty acids (alpha-linolenic acid (C18:3n3), eicosatrienoic acid (C20:3n3), gamma-linolenic acid (C18:3n6), and docosadienoic acid (C22:2) [8]. More importantly, another alternative option to reuse the waste is to process the frames with enzymatic hydrolysis and powder them into tilapia hydrolysate powder (THP). In our previous work, regarding the combination of proteomic techniques and in silico analysis, enzymatic hydrolysis can regenerate and change the functional and physicochemical attributes of the food product. Subsequently, there is an idea to carry on the nutritive value of the hydrolyzed proteins and make healthier products. The high nutritional value of the hydrolysates was shown by their protein contents and amino acid profiles [9,10]. In addition, an in vitro assay of the hydrolysates and peptide fractions demonstrated varying bioactivities, including ACE inhibitory, DPPH radical scavenging, reducing power, and antibacterial activities [11].

To conclude, SBs can be important vehicles for transporting these ingredients and providing bioactive compounds to the human diet [12]. Consequently, this paper aimed to evaluate tilapia fish by-products as a novel food ingredient to analyze different physicochemical and functional properties among SBs enriched with TDP and THP. Meanwhile, considering the importance of cooking method diversification on enriched food products, the effects of different cooking methods, baking and no baking, on the physicochemical and functional properties (including the antioxidant, ACE inhibitory, and antibacterial activities) of the enriched SB production were investigated and compared.

## 2. Materials and Methods

### 2.1. Materials

Tilapia *(Oreochromis niloticus)* frames were collected for research purposes from a local seafood processing plant (Fortune Life Company, Kaohsiung, Taiwan) and kept in −20 °C until further use. Bromelain enzyme specialty for food and beverages was purchased from Amano enzyme Inc. (Nagoya, Japan). For SB production, all of the materials were commercial products composed of whole oat (Quaker), fine oat flakes (Quaker), rice flakes (Kellog’s), jumbo raisins (Trygood’s—beans group), Fructose (Fong Leng), and crystal sugar. Two commercial snack bars, Nestle (Brand A) and Nature Valley (Brand B), were used to compare with the developed bars. For chemical materials, ACE from rabbit lung (≥2 units/mg protein) and the substrate N-(3-[2-furyl]acryloyl)-phenylalanylglycylglycine (FAPGG) and DPPH (2,2-diphenyl-1 picrylhydrazyl were purchased from Sigma Aldrich. Microorganisms were initially purchased from Bioresource Collection and Research Center (BCRC), Taiwan. Escherichia coli BCRC 10675 and Staphylococcus aureus BCRC 10780 cultures were prepared from the Microbiology Laboratory, Department of Food Science, NTOU. Muller Hinton agar was prepared for antibacterial activity analysis by combining Mueller Hinton Broth and 1.5% Bacto Agar from Difco Culture Media (Franklin Lakes, NJ, USA). Other used chemicals and reagents were of analytical grade.

### 2.2. Preparation of Tilapia by-Product Dry Powder (TDP)

Tilapia frames were washed with water and cleaned to minimalize the contamination during transportation and handling. Then, the frames were steam-cooked for about 10–20 min to remove the meat that adhered to the bones. After completing the cooking process, the meats and the frames were then heat-treated with the frying pan for 1–2 min to remove excess moisture. The cooked meats and frames were ground and dried in a hot air oven at 60 °C for 12 h with water activity (Aw) measure at 0.3. The dried frames were powdered using a mixer and packed in airtight containers [13].

### 2.3. Preparation of Tilapia by-Product Hydrolysate Powder (THP)

The tilapia by-product hydrolysate powder (THP) was made with thaw-minced tilapia by-product (frames), which thawed overnight in a cold room (4 °C), then 15% *w/v* of minced sample was taken in the beaker. An equal volume of distilled water was added, and the mixture was cooked for 30 min (to inactivate the enzyme in the raw material), and further cooled to 55 °C, and pH was adjusted to 6.5–6.8 using 1N HCl. After that, 0.5% bromelain enzyme (Amano enzyme Inc., Japan) was added to the cooked sample, which was then kept in a water bath. Moreover, the hydrolysis reaction was continued at 55 °C for 45 min. The hydrolyzed mixture was heated up to 80 °C for 15 min, and the aqueous portion was separated by filtration and centrifugation (2000× *g*). The residue was insoluble matter during the hydrolysis process and was discarded. Finally, the aqueous hydrolysate was subjected to freeze-drying. The freeze-dried tilapia frame hydrolysates were stored in an airtight container until further use.

### 2.4. Preparation of Snack Bars (SBs)

**Figure 1:**
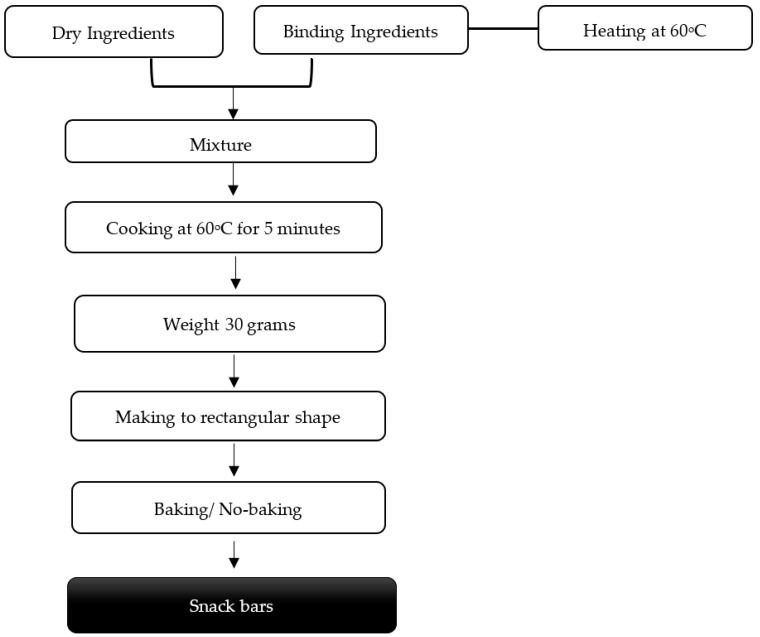
Snack bar development flow chart.

Snack bars (SBs) were prepared using tilapia by-product dry powder (TDP) and tilapia hydrolysate by-product powder (THP) supplementation. The SB formulation was adapted from the reported SB recipe with some modifications [14]. The SB formulation (Table 1) was previously tested for complete agglomeration of solid ingredients. The details of the snack bars processing method are shown in Figure 1. Binding ingredients (fructose, crystal sugar, and raisins) and dry ingredients were mixed and heated at 60 ± 3 °C. The dough was made into a rectangular shape with aluminum molds. Two common ways of processing were used, baking and no baking (cooling at room temperature). 

The processing approaches of SB formulations were cooked and baked or cooled at room temperature. Each SB formulation was homogenized and mixed well. Nutritional values, including the moisture, lipid, protein, and ash contents, were determined by AOAC methods. Carbohydrate content was estimated as the difference between 100% and the sum of the moisture, protein, ash, and lipid contents. Energy value was calculated as energy value (kcal/100 g) = 4 × protein% +9 × lipid% + 4 × carbohydrate% [15].

### 2.5. Color Determination

L*, a*, and b* color parameters were analyzed using Tokyo Denshoku TC-1800MK-II Colorimeter (Shinjuku, Tokyo, Japan). Additionally, chromaticity coordinates (a* and b*) were used to calculate chroma (C*) and hue angle (H°). The equation determined the total color difference (ΔE) between enriched SBs formulations and control group (without tilapia powder addition):

ΔE = [(ΔL*)^2^ + (Δa*)^2^ + (Δb*)^2^]^1/2^

### 2.6. Texture Measurement

Texture measurements, including the hardness, brittleness, and stiffness, were performed using a TA-HD texture analyzer XT-RA (Stable Micro Systems, Vienna Court, Surrey, United Kingdom) with a 10 kg load cell and crosshead speed of 1.67 mm/s. Brittleness was measured as the initial fracture distance (mm). Stiffness was calculated as breaking force divided by distance [16].

### 2.7. Angiotensin I Converting Enzyme (ACE) Inhibitory Activities Test

Angiotensin I converting enzyme (ACE) inhibitory activities were measured by using N-[3-(2-furyl) acryloyl]-L-phenylalanyl glycyl glycyl (FAPGG) as the synthetic substrate for ACE. The modified method was based on the combination of reported assays from [17,18]. FAPGG and the sample were prepared in 50 mM Tris-HCl buffer to contain 0.3 M NaCl and adjusted to pH 7.5. A 170 µL aliquot of 0.5 mM FAPGG was mixed with 10 µL of ACE (0.5 U/mL, last activity of 25 mU) and 20 µL of sample. The decreased absorbance at 345 nm was measured at regular intervals (every 3 min) for 30 min at 37 °C using a Synergy H4 microplate reader (Biotek Instruments, Winooski, VT, USA). Tris-HCl buffer was used instead of sample solution as a control. ACE activity was expressed as the rate of reaction (ΔA/min), and inhibitory activity was calculated as follows:

ACE inhibition (%) = [1−ΔAmin−1(sample)/ΔAmin−1(control)] × 100%

where ΔAmin−1(sample) and ΔAmin−1(control) are ACE activity in the presence and absence of the peptides, respectively.

### 2.8. DPPH Radical Analysis

DPPH radical analysis was determined based on the combination of the previous methods used by [19,20]. The sample was first prepared by dissolving 5 mg of SBs in 5 mL of 0.1 M sodium phosphate buffer (pH 7). One hundred microliters of the sample was mixed with 100 µL of DPPH (0.1 mM, dissolved in methanol) in a 96-well plate and incubated in a dark place for 30 min. The absorbance was measured by using multiple readers (Multiskan Go, Thermo Fisher Scientific, Waltham, MA, USA) at 517 nm. Double distilled water (ddH2O) was used for the control sample, and vitamin C was used as the positive control. Ultimately, DPPH radical scavenging activity was calculated using the following equation:

DPPH radical scavenging activity (%) = ((Abs.control−Abs.sample)/(Abs.control)) × 100%

### 2.9. Microbiological Test

Previously, defatted SBs were dissolved first with dimethyl sulfoxide (DMSO) solution according to the desired concentration (1 mg/mL) with three replications at each concentration [21]. The Kirby–Bauer disk diffusion method was applied to measure the antibacterial activity of the collected samples. Lawns of two bacterial test suspensions (*Escherichia coli* BCRC 10675 and *Staphylococcus aureus* BCRC 10780 cultures) were prepared using the log-phase cells (the culture turbidity was compared to the 0.5 McFarland standard equivalent to 105 cells/mL) on the Mueller–Hinton agar (MHA). The wells were prepared with a borer on the MHA. The corresponding wells were injected with 10 mg/mL of the crude samples. Negative controls used buffered sterile peptone water, and positive controls used chloramphenicol (10 μg). After the plates were then incubated at 37 °C for 15 h, the observation was done by evaluating the appearance of inhibition zones [22].

### 2.10. Statistics Analysis

The data were processed and analyzed with the statistical software SPSS version 25.0.0 for Windows (SPSS, Chicago, IL, USA). Analytical determinations for the samples were performed in triplicate, and standard deviations were reported. Tukey’s test ascertained a comparison of the means at a 5% significance level by analysis of variance (ANOVA). Pearson correlations were used to correlate the physicochemical properties. Principal component analysis (PCA) was carried out to evaluate the relationships among the studied properties and visualize the similarities between them.

## 3. Results and Discussion

### 3.1. Physical Properties

#### 3.1.1. Appearance and Color

The effect of substitution of 10% tilapia by-products powders on the physical quality of SBs was presented in Table 2. The addition of 10% tilapia hydrolysate powder (THP) increased the weight in the baked group. Also, with the addition of THP, there was not much change in the length, width, though concerning thickness had a slight increase in the SBs. However, with the addition of TDP, the weight decreased both in the baked and unbaked processing methods. There was not much change in the length of the bars, though a slight increase in width and thickness of the SBs was observed.

**Figure 2:**
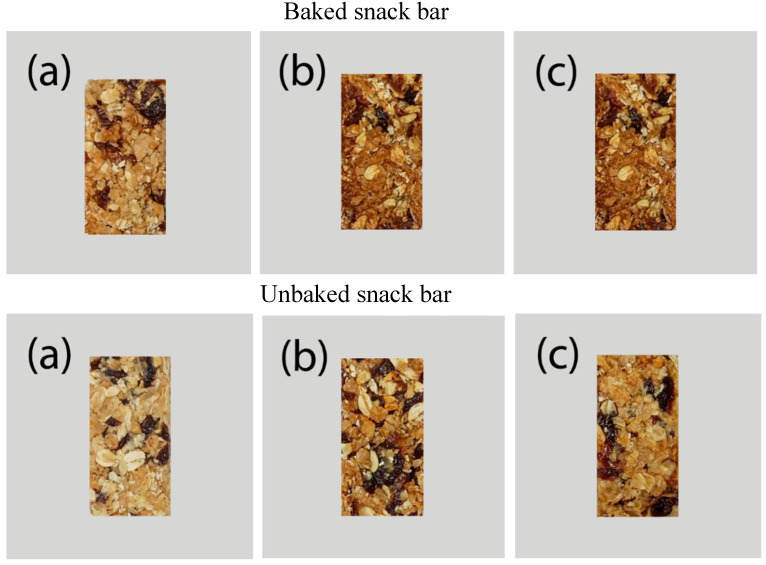
Photograph of snack bars with different substitutions of tilapia by-product powder. (**a**): original snack bars/ control (SB); (**b**): snack bars with tilapia dry powder (SB + TDP); (**c**): snack bars with tilapia hydrolysate powder (SB+ THP).

Color contributes to the quality of food and the attraction to consumers. The Maillard reaction is primarily responsible for color development when most foods are heated. The cooking process develops flavor, aroma, and color due to the browning effect and the formation of colored compounds from the Maillard reaction on the food surface [23]. The crust color changed from golden brown to slightly dark brown with TDP substitution and THP. The surface was smooth in the control (without TDP and THP) group. It was slightly rough in the treatment groups (Figure 2). According to Table 3, the cooking method significantly affected the color of baked and unbaked SBs. The baked sample had yellow coloration, was darker (lower L* value), and had more reddish color (higher a* value) than the unbaked sample. That is, darkening may be related to the production of compounds formed in the caramelization reaction. The caramelization reaction is related to the production of browning compounds in foods, which occurs prior to sucrose hydrolysis at high temperatures [24].

#### 3.1.2. Hardness Test

The results presented in Table 4 showed that the addition of tilapia hydrolysate powder (THP) significantly improved the hardness of the SBs. This result agrees with those studies that reported the addition of shrimp hydrolysate powder with increased hardness of the extrudate snacks, resulting in the compact structure and less expansion of the product [13]. These changes may be due to protein interactions at a higher level. As baking started and the temperature of the bar dough increased, the pores were filled up, as mentioned before, and the product tended to behave more like a solid, increasing the springiness to the maximum. Additionally, as baking progressed, the water evaporated from the surface, and crust formation occurred, leading to increased hardness. Moreover, as dough viscosity changed due to the gelatinization of starch and denaturation of protein, new gas cells were formed, the dough structure became too weak to withstand the compressive force, and stiffness started to decline [25].

### 3.2. Nutritional Properties

The proximate composition of SB products is given in Table 5. Increased protein content from the addition of both TDP and THP was observed treatment groups. Similar studies indicated that the addition of tilapia dry powders was used to increase the protein levels and improve the quality of some foods such as bread [26], pasta [27], and instant soup [28]. However, the THP treatment group had the most protein. The result has not previously been described. Tejano et al. reported that the hydrolysates had high nutritional value reflected by their protein contents and amino acid profiles. Additionally, the hydrolysates and peptide fractions demonstrated various bioactivities [11].

Concerning fat of the SBs, crude fat content (29.56 ± 0.55%) of the TDP treatment group was higher than that of the control and the THP treatment group (*p* < 0.05). The World Health Organization recommends that the energy content of a daily balanced diet be composed of a variable contribution of 55–57% carbohydrates, 15–30% fat, and 10–15% protein. To elaborate, the difference in crude fat content significantly impacted the total calories of SBs. Lower calorie content is a desirable attribute for consumers because consuming a high-fat diet (HFD) has been increasingly viewed as a significant modifiable risk factor for diabetes and certain types of cancer [29].

Regarding carbohydrates, their content in TDP and THP groups was significantly lower than in the control SB (*p* < 0.05). Traditionally, carbohydrates were the major compounds in SBs. However, adding both TDP and THP could provide the SBs with fewer carbohydrates. In this sense, replacing at least partially high-calorie ingredients traditionally used in the baking industry could be a strategy to meet the calorie reduction requirements [16]. Additionally, the difference of saturated fatty acid production influenced the different amounts of energy content in food. It usually occurred in some food replacement or supplementation products [30]. These findings would support that SB supplementation has potential for substantial reductions in total energy intake [31].

The correlation between physicochemical properties of SBs is shown in Table 6. L* (lightness) was negatively correlated to a* (redness) and positively correlated to b* (yellowness). This suggested that the higher lightness of the SBs, the higher the redness and the lower yellowness. Stiffness was observed to be negatively correlated to brittleness and positively correlated to width, yellowness, and hardness. The hardness of the SBs was found to increase in width and yellowness. Moisture positively correlated with brittleness and negatively correlated with crude protein content. Additionally, the higher crude fat content indicated higher ash and carbohydrate content.

### 3.3. Functional Properties

**Figure 3:**
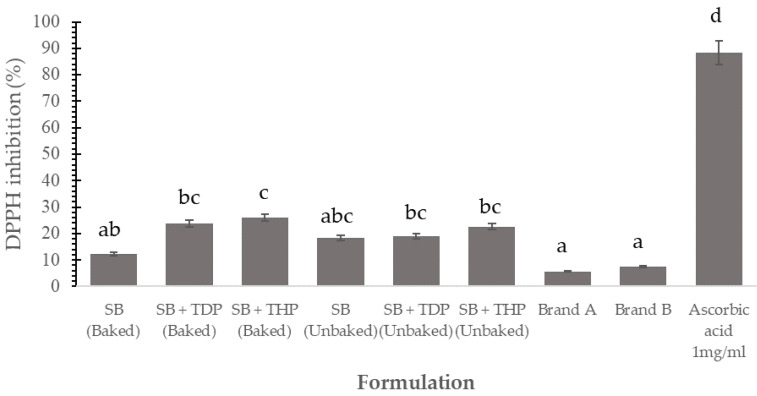
DPPH scavenging activity of snack bar products. Values that contain different letters are Scheme 0.

Radical scavenging activity measures antioxidant activity. The compound 2,2-diphenyl-1-picrylhydrazyl (DPPH) is a stable free radical and widely used to measure the radical scavenging activity of different bioactive constituents. DPPH radicals affected by the snack bar antioxidants are shown in Figure 3. The control group showed the lowest activity, followed by the SB + TDP and SB + THP with the highest activity (*p* < 0.05), with DPPH scavenging activity ranging from 12.40 to 26.04%. Consequently, both TDP and THP can be used as functional ingredients to perform the antioxidant activity in food products. The applied baking condition had an excellent potential to release phenolic compounds associated with dietary fiber because DPPH scavenging activity was not significantly affected by the temperature [32]. Ambigaipalan and Shahidi also found that muffins incorporated with date seed flour hydrolysate significantly increase the DPPH scavenging activity compared to that in control muffins [33]. The higher antioxidant activity is predicted from the physical and chemical changes in the microstructure of the food products. This increased antioxidant activity may be caused by the liberation of high amounts of antioxidant components due to the thermal destruction of cell walls and subcellular compartments. It was also predicted during the thermal and chemical reaction from producing different radical-scavenging antioxidants, inactivation of oxidative enzymes, and new formation of novel compounds in the Maillard reaction [34].

**Figure 4:**
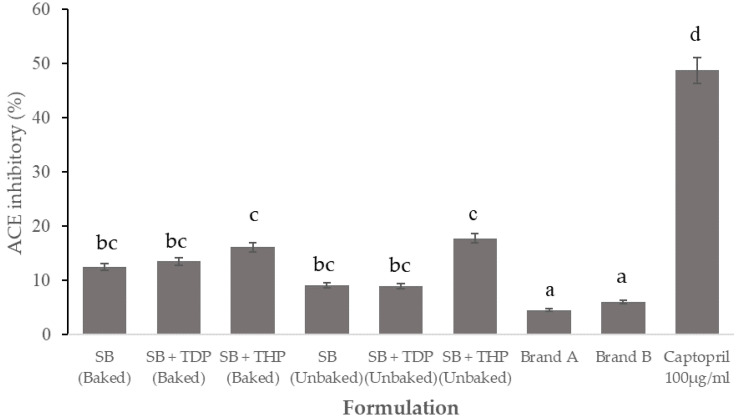
ACE inhibitory activity of snack bar products. Values that contain different letters are significantly different at *p* < 0.05.

ACE inhibitory activities were evaluated with SBs that were prepared by incorporation of tilapia dry powder (TDP) and tilapia hydrolysate powder (THP). In Figure 4, both the SB + TDP and SB + THP had increased significantly in the ACE inhibitory activity than the control group. Furthermore, the baking process of the SB + TDP and SB + THP significantly increased the SB’s ACE inhibitory activity. Bioactive components could be generated from decomposed oat proteins and tilapia powders (TDP and THP) during the thermal processing, leading to ACE inhibitory activities. This experimental result was in agreement with our previous works where bromelain was used as the proteinase leading to the release of the peptide with ACE inhibitory activity from tilapia by-products [35]. Further study demonstrated the rapid reduction in blood pressure and a long duration of the antihypertensive effect, which indicated that the protein hydrolysate from the tilapia frame was able to develop into a promising ingredient for the formulation of antihypertensive functional food [36]. Tavares et al. reported that whey protein hydrolysate had a hypotensive effect in rats. This finding confirms that bioactive peptides, including ACE inhibitors, may generally be obtained from food protein by hydrolysis [37]. On the other hand, the enzymatic release of peptides from parent proteins is possible during (a) the process of protein digestion in the GI tract, (b) food processing, and (c) proteolysis by enzymes of microorganism and plant origin [38]. As a result, this study demonstrated that tilapia hydrolysate powder could be used as an ACE inhibitor in SB products.

### 3.4. Microbiological Analysis

Table 7 shows that all samples exhibited antibacterial activity against *Staphylococcus aureus* BCRC 10780 (Gram-positive bacteria), with the SB + THP giving the highest activity to inhibit bacterial growth. Generally, the hydrolysate powder is well-known for having antibacterial peptides to inhibit bacterial growth. However, the SB products could not inhibit *Escherichia coli* (Gram-negative bacteria). A similar result was reported that the yellowfin tuna hydrolysates possessed higher antibacterial activity on Gram-positive than Gram-negative bacteria [39]. Peptides have different modes of action with a broad-spectrum against Gram-positive and Gram-negative strains, which are related to the ability of these peptides to interact with the bacterial membrane and their ability to disrupt the metabolism of the bacteria. Gram-positive bacteria have cell walls composed of a thick layer of peptidoglycan, whereas Gram-negative bacteria have only a layer of lipopolysaccharide at the external surface, followed by a thin layer of peptidoglycan [40].

### 3.5. Principal Component Analysis (PCA)

PCA is a statistical mathematical tool to identify variation present in the dataset, usually to characterize the samples using a small number of factors. In this study, Principal Component Analysis was analyzed attributed to four components that explained total variation (Table 8). The four factors explained 83.73% of the variation in the total SBs quality.

The principal component (PC) were labeled based on the SBs properties that loaded as follows:

1. PC 1: nutraceutical pigmentation (L*, a*, b*, DPPH scavenging, and ACE inhibitory) with the highest contribution to the selected quality parameters (30.16%).
2. PC 2: physical characteristics (weight, thickness, length, width, hardness, and stiffness) contributed to the quality 24.02%.
3. PC 3: nutrition value (crude protein, crude fat, and carbohydrate) with the contribution to quality of 17.49%.
4. PC 4: greater dehydration (moisture and brittleness) with the contribution to quality of 12.07%.

The results of the current investigation were in line with the finding that investigated the principal component physicochemical and nutraceutical in guava products [42]. Nutraceutical pigmentation (the correlation of color changes and biological activity) highly contributed to the quality of the snack bars. The addition of THP and TDP that provided biological activities that made color changes in the products. Particularly during the processing, the biological activities change, and the color changes of the products can be the indicator.

### 3.6. Sensory Evaluation

Sensory evaluation of the developed SBs was performed and are described in the supplementary file. Sensory attributes are defined in Table S1. As shown in Table S2, SB (control) obtained the highest score at weight mean value (WMV) both in the baked group (3.63) and the unbaked group (3.39), followed by the SB + TDP with the WMV of 2.81 for the baked group and 2.59 for the unbaked group. SB + THP WMV was 2.65 for the baked group and 2.66 for the unbaked group. These properties drove quality for the experts that were determined depending on the scores:

1. Unacceptable (<2.5)
2. Good (2.5–3.5)
3. Very good (3.5–4.5)
4. Excellent (>4.5).

The point-based method used to evaluate the sensory evaluation of the properties of the snack bars showed that the best score (3.5–4.5, very good quality) was obtained for the samples SB (control) in the baked group, and other treatments had a good score (2.5–3.5 good quality).

### 3.7. Storage Analysis

Storage analysis including sensory evaluation (supplementary method 1) and microbiological analysis (supplementary method 2) was also investigated within a three-months period, and the experimental results are shown in Table S1 and S2. In Figure S3, the water activity (Aw) value of the unbaked group was higher than that in the baked group, with the final value of Aw for the baked group being 0.41 (SB), 0.41 (SB + TDP), and 0.44 (SB + THP), respectively. Changes in moisture content occurred during storage time (Figure S4). The moisture content of the snack bars increased slightly during the three months of storage. According to Figure S1, S2 and S3, SB + THP had lowest number of the bacteria, yeast, and mold growth compared with those of SB and SB + TDP. This result was in line with the antibacterial effect of the snack bars that could inhibit *Staphylococcus aureus*, the most common bacteria in food processing. Based on the present investigation in Figure S5, the increase of color changes during three months of storage with the baked group had a higher increase than the unbaked ones. This predicted that the color change could indicate the extent of the Maillard reaction in the food system.

## 4. Conclusions

In this study, SBs were prepared with the addition of tilapia dry powder and tilapia hydrolysate powder. From the physical point of view, baked SBs had higher hardness than unbaked SBs. However, baked SBs showed darker colors because of caramelization’s browning compounds. The addition of these materials to SBs enhanced the nutritional value of the products by increasing the protein and fat content and influence the energy produced. In these SBs, all samples exhibited potential for DPPH scavenging activity, ACE inhibitory activity, and antibacterial activity. In particular, SB + THP showed the highest activity. More functional properties were observed in SB + THP because of the bioactive peptides from THP. However, SB + TDP was still recommended for production due to the convenience of preparation with good functional properties. Principal component analysis reported that physicochemical and functional properties contributed 83.73% to overall quality and were separated into four principal components: nutraceutical pigmentation, physical characteristics, nutritional value, and greater dehydration. It is suggested that tilapia by-product powders (both TDP and THP) can be alternative options for adding nutraceutical values to food products.

## Figures and Tables

**Table 1:** Formulation of snack bars.

Ingredients	SB	SB + TDP *	SB + THP *
Rice Flakes (g)	13.72	12.35	12.35
Whole oat Flakes (g)	18.75	16.87	16.87
Thin Oat Flakes (g)	19.00	17.09	17.09
Tilapia by-product dry powder (TDP) (g)	0	5.14	0
Tilapia hydrolasate by-product powder (THP) (g)	0	0	5.14
Raisins (g)	29.30	29.30	29.30
Fructose (g)	28.46	28.46	28.46
Crystal Sugar (g)	26.03	26.03	26.03
Lemon Water (g)	5.00	5.00	5.00

Source: [14] with slight modification. *: Amount of TDP and THP added was 10% of the dry ingredients (rice flakes, whole oat flakes, thin oat flakes, and TDP/THP) in grams.

**Table 2:** Effect of different substitution of tilapia by-product powder on physical quality of snack bars.

Formulation	Weight (g)	Length (mm)	Width (mm)	Thickness (mm)
SB (Baked)	28.31 ± 0.09 ^c^	67.17 ± 0.72 ^a^	34.2 ± 0.89 ^cde^	15.96 ± 1.24 ^b^
SB + TDP (Baked)	27.32 ± 0.28 ^bc^	67.18 ± 0.36 ^a^	35.22 ± 0.18 ^de^	19.05 ± 0.21 ^c^
SB + THP (Baked)	28.85 ± 1.16 ^c^	66.53 ± 0.43 ^a^	35.24 ±1.05 ^de^	15.65 ± 0.19 ^b^
SB (Unbaked)	29.13 ± 0.84 ^c^	66.05 ± 0.05 ^a^	32.42 ± 0.57 ^bc^	16.86 ± 0.25 ^bc^
SB + TDP (Unbaked)	27.39 ± 0.02 ^b^	65.61 ± 0.33 ^a^	31.72 ± 0.13 ^b^	16.95 ± 0.13 ^bc^
SB + THP (Unbaked)	30.02 ± 0.33 ^c^	65.9 ± 0.17 ^a^	33.49 ± 0.26 ^bcd^	16.55 ± 0.05 ^bc^
Brand A	23.91 ± 0.04 ^ab^	92.12 ± 0.15 ^b^	29.54 ± 0.28 ^a^	17.53 ± 1.28 ^bc^
Brand B	21.86 ± 0.06 ^a^	89.78 ± 0.14 ^b^	36.15 ± 0.02 ^e^	9.20 ± 0.40 ^a^

Data are presented as mean ± standard deviation with triplicate measurements. For each column, values that contain different letters in superscript are significantly different at *p* < 0.05.

**Table 3:** Color analyses of snack bar products.

Formulation	L*	a*	b*	∆E
SB (Baked)	66.38 ± 0.76 ^c^	1.06 ± 0.48 ^d^	42.39 ± 0.60 ^c^	
SB + TDP (Baked)	58.71 ± 0.27 ^b^	1.00 ± 0.47 ^d^	41.72 ± 0.93 ^b^	9.41 ± 1.94
SB + THP (Baked)	76.12 ± 1.32 ^d^	−1.26 ± 0.55 ^c^	42.09 ± 0.15 ^d^	11.36 ± 0.84
SB (Unbaked)	74.18 ± 0.82 ^d^	−6.36 ± 0.44 ^b^	39.09 ± 0.41 ^b^	
SB + TDP (Unbaked)	92.36 ± 0.75 ^e^	−10.55 + 0.32 ^a^	37.99 ± 0.90 ^c^	19.06 ± 1.47
SB + THP (Unbaked)	39.92 ± 0.20 ^a^	7.07 ± 0.41 ^f^	30.89 ± 0.94 ^a^	38.25 ± 1.69
Brand A	57.20 ± 0.56 ^b^	3.58 ± 0.06 ^e^	47.91 ± 0.36 ^c^	
Brand B	66.54 ± 0.29 ^c^	3.97 ± 0.20 ^e^	51.07 ± 0.04 ^d^	

Data are presented as mean ± standard deviation with triplicate measurements. For each column, values that contain different letters in superscript are significantly different at *p* < 0.05.

**Table 4:** Texture analyses of snack bar products.

Formulation	Hardness (N)	Stiffness (kg/m)	Brittleness (mm)
SB (Baked)	25.14 ± 0.69 ^c^	1427.36 ± 0.34 ^bc^	17.6 ± 0.91 ^a^
SB + TDP (Baked)	24.27 ± 0.29 ^bc^	1327.70 ± 0.20 ^bc^	18.37 ± 0.80 ^a^
SB + THP (Baked)	27.39 ± 0.43 ^c^	1997.37 ± 0.23 ^cd^	15.22 ± 0.55 ^a^
SB (Unbaked)	12.43 ± 0.21 ^a^	677.48 ± 0.14 ^ab^	18.43 ± 1.03 ^a^
SB + TDP (Unbaked)	10.46 ± 0.46 ^a^	544.89 ± 0.23 ^a^	19.16 ± 0.19 ^a^
SB + THP (Unbaked)	13.35 ± 0.11 ^ab^	787.10 ± 0.16 ^ab^	17.43 ± 0.66 ^a^
Brand A	10.79 ± 0.21 ^a^	556.14 ± 0.15 ^a^	19.66 ± 1.43 ^b^
Brand B	29.83 ± 0.41 ^c^	2720.66 ± 0.50 ^d^	11.03 ± 0.61 ^a^

Data are presented as mean ± standard deviation with triplicate measurements. For each column, values that contain different letters in superscript are significantly different at *p* < 0.05.

**Table 5:** Proximate analyses of snack bar products.

Material	Baked			Unbaked		
	Control	SB TDP 10%	SB THP 10%	Control	SB TDP 10%	SB THP 10%
Moisture (%)	3.90 ± 0.57 ^a^	3.90 ± 0.52 ^a^	3.09 ± 0.47 ^a^	3.86 ± 0.28 ^a^	3.85 ± 0.28 ^a^	3.24 ± 0.29 ^a^
Crude Protein (%)	23.91 ± 0.57 ^a^	27.41 ± 0.57 ^b^	32.08 ± 0.57 ^c^	21.58 ± 0.57 ^a^	27.41 ± 0.57 ^b^	32.08 ± 0.52 ^c^
Crude Fat (%)	3.12 ± 0.03 ^a^	29.56 ± 0.55 ^b^	9.61 ± 0.03 ^a^	3.30 ± 0.03 ^a^	24.13 ± 0.24 ^b^	7.71 ± 0.18 ^a^
Ash (%)	1.75 ± 0.62 ^a^	2.78 ± 0.14 ^a^	2.14 ± 1.17 ^a^	1.65 ± 0.55 ^a^	3.15 ± 1.33 ^a^	1.69 ± 0.63 ^a^
Carbohydrate (%)	66.45 ± 0.02 ^c^	36.74 ± 0.19 ^ab^	53.07 ± 0.03 ^bc^	69.61 ± 0.03 ^c^	17.32 ± 0.14 ^a^	55.27 ± 0.10 ^bc^
Energy (kcal)	389.64 ± 2.46 ^a^	519.10 ± 8.36 ^b^	427.13 ± 4.01 ^ab^	394.48 ± 2.77 ^a^	492.66 ± 6.50 ^ab^	418.79 ± 4.86 ^ab^
Water Activity (Aw)	0.33 ± 0.22 ^b^	0.29 ± 0.01 ^a^	0.39 ± 0.01 ^d^	0.35 ± 0.01 ^bc^	0.36 ± 0.01 ^c^	0.43 ± 0.01 ^e^

Data are presented as mean ± standard deviation with triplicate measurements. For each row, values that contain different letters in superscript are significantly different at *p* < 0.05.

**Table 6:** Pearson coefficient correlation of physicochemical properties of snack bar products.

Parameter	WE	LE	WI	TH	L*	a*	b*	HA	BR	ST	P	F	M	A	C
WE	1														
LE	−0.28	1													
WI	−0.49	0.81 **	1												
TH	−0.42	0.53	0.15	1											
L*	−0.44	−0.17	−0.35	−0.15	1										
a*	0.39	0.26	0.57	−0.01	−0.94 **	1									
b*	−0.37	0.24	0.23	−0.29	0.77 **	−0.53 *	1								
HA	−0.13	0.55	0.76 **	−0.27	−0.07	0.32	0.49*	1							
BR	−0.02	−0.32	−0.44	0.17	0.11	−0.27	−0.24	−0.28	1						
ST	−0.08	0.55	0.78 **	−0.31	−0.05	0.33	0.54 *	0.96 **	−0.52 *	1					
P	0.31	−0.03	0.35	−0.14	−0.32	0.44	−0.13	0.22	−0.35	0.32	1				
F	−0.59 *	0.14	−0.13	0.61*	0.46	−0.49 *	0.14	−0.35	0.25	0.37	0.12	1			
M	−0.40	0.32	0.13	0.46	0.25	−0.35	0.02	−0.20	0.52 *	−0.35	−0.54 *	0.31	1		
A	−0.17	0.01	−0.11	0.22	0.34	−0.34	0.18	0.00	0.21	−0.05	0.18	0.64 **	0.23	1	
C	0.50	−0.13	0.51	−0.55	−0.37	0.38	−0.10	−0.25	−0.19	0.26	−0.30	0.97 **	−0.20	−0.72	1

(*) indicates significance at the 0.05 level (two-tailed); (**) indicates significance at the 0.01 level (two-tailed); WE: weight; LE: length; WI: width; TH: thickness; L*: lightness; a*: redness; b*:yellowness; HA: hardness; BR: brittleness; ST: stiffness; P: protein; F: fat; M: moisture; A: ash; C: carbohydrate.

**Table 7:** Antibacterial activity of snack bar products.

Samples	Growth Inhibition Zone Diameter (GIZD) in mm *	
	*E. coli* BCRC 10675	*S. aureus* BCRC 10780
SB (Baked)	-	10.23 ± 1.64 ^a^
SB + TDP (Baked)	-	10.01 ± 0.28 ^a^
SB + THP (Baked)	-	10.99 ± 0.62 ^a^
SB (Unbaked)	-	8.52 ± 0.62 ^a^
SB + TDP (Unbaked)	-	8.64 ± 0.31 ^a^
SB + THP (Unbaked)	-	15.08 ± 1.95 ^b^
Chloramphenicol	+	+

* The value represents averages ± standard deviations for triplicate experiments (*n*= 3). Different superscript letters represent significant differences (*p* < 0.05) among the activities of the samples; -: no inhibition; +: maximum inhibition.

**Table 8:** Score coefficients derived from principal component analysis of snack bars product quality.

Variable	PC1	PC2	PC3	PC4	Communalities
Weight	0.39	**−0.52**		0.27	1
Thickness	−0.27	**0.77**	0.27	−0.31	1
Length	0.34	**0.70**	0.26	−0.36	1
Width	0.39	**0.62**	0.11	−0.27	1
L* (lightness)	**−0.69**	0.39	−0.36	0.17	1
a* (redness)	**0.83**	−0.19	0.39	−0.14	1
b* (yellowness)	**−0.68**	0.18	−0.38		1
Hardness	0.39	**0.70**	−0.26		1
Stiffness	0.34	**0.72**	−0.37		1
Brittleness	−0.37	0.19	0.23	**−0.72**	1
Moisture	−0.37	0.19	0.23	**−0.72**	1
Crude Protein	−0.32	−0.37	**0.50**		1
Crude Fat	−0.36	0.25	**0.47**	0.27	1
Ash	**−0.52**	0.33	0.25	0.37	1
Carbohydrate	0.31	−0.25	**−0.50**	−0.40	1
DPPH scavenging	**0.49**	0.33	0.32	0.29	1
ACE inhibitory	**0.85**	0.27	0.20	0.21	1
Proportion Variance	30.16%	24.02%	17.49%	12.07%	
Cumulative Variance	30.16%	54.1%	71.66%	83.73%	

Extraction method: principal component analysis; rotation method varimax with Kaiser normalization. Score coefficients >0.4 are shown in bold [41].

## Data Availability

The datasets generated for this study are available upon request to the corresponding author.

## Supplementary Materials

The following are available online at https://www.mdpi.com/article/10.3390/foods10081908/s1, Supplementary Method 1: Sensory evaluation, Supplementary Method 2: Storage analysis, Figure S1: Bacteria number of snack bars during storage, Figure S2: Mold number of snack bars during storage, Figure S3: Water activity of snack bars during storage, Figure S4: Moisture content of snack bars during storage, Figure S5: Color changes (∆E) of snack bars during storage, Table S1: Sensory attributes and their definitions, Table S2: Sensory evaluation of snack bars.
