# Supplementary material for: Physicochemical and Functional Properties of Snack Bars Enriched with Tilapia (Oreochromis niloticus) By-Product Powders
Source: Foods. 2021 Aug 17;10(8):1908. doi: 10.3390/foods10081908 (PMC8392232; doi:10.3390/foods10081908)
Supplement: Supplementary file 1 [file foods-10-01908-s001.zip › foods-1280624-supplementary.pdf]

Supplementary Methods

1. Sensory evaluation

Sensory evaluation was carreid out with 86 palnelists of graduate students from National Taiwan Ocean University. Senroy attributes and their definitions is shown in Table S3. The overall acceptance of the SBs product was measured the consumers' like or accept SBs as new product developed. Scoring method that used to assess the SBs products was 5-point structured hedonic scale ranging from 1 (highly disliked) to 5 (highly liked). The sensory attributes were using the similar confectionery products assessment in the previous studies [1-4] .

2. Microbiological analysis during storage

The microbiological for safety test was using the method of USDA (2001) with slightly modification. Total plate count (TPC) for the bacteria and mold were measured at 3 months of storage at ambient condition or (30 ± 2 °C) temperature. 25 gram of sample was aseptically weighed and put in a stomacher bag with 250 mL of 0.1% peptone buffer and agitated for 120 s after closing the bag. After that, 0.1 mL solution was placed on plate count agar (for the bacteria) and Dicloran Glycerol (DG-18) Agar Base for mold. All of the media were provide by Neogen Culture Media (Lansing, MI, USA). For the bacteria, the plates were incubated at 37 ± 1°C for 48 h. The mold plates were incubated upside down at 25°C for 5-7 days. The number of CFU were counted and reported as log CFU/g. The analysis was conducted in triplicates.

Supplementary Table S1

Table S1. Sensory attributes and their definitions

Sensory attribute	Definition
Overall acceptance	Degree of acceptance of the products
Texture attributes	
Hardness	Degree of the complex feeling when bites
Crispness	Degree of the crispy feeling when bites
Lumpiness	Chew five times on sample on the sample and evaluate to which degree the sample crumb forms into a lump
Moistness	Take a bite of the sample e and evaluate how much it wet your mouth
Flavor attributes	

Salty flavor	The intensity of salty flavor/ perceived salty flavor of the sample evaluated in the mouth.
Spicy flavor	The intensity of spicy flavor/ perceived spicy flavor of the sample evaluated in the mouth
Butter flavor	The intensity of butter flavor/ perceived butter flavor of the sample evaluated in the mouth.
Overall taste	Degree of deliciousness
Greasiness	Degree of the oily feeling in the mouth
Odor	Degree of the odor with the foul smell
Rancid odor	Degree of the odor of rancid fat
Color attributes	
Color difference	Degree of color difference compared with the control product
Lightness difference	Degree of the lightness difference compared with the control product.

Source: [1], [2], [3], and [4]

Table S2. Sensory evaluation of snack bars

	Baked			Unbaked		
Sensory attributes	SB	SB+TDP	SB+THP	SB	SB+TDP	SB+THP
Overall acceptance	4.05 ± 0.84[c]	2.78 ± 0.99[b]	2.41 ± 1.00[ab]	3.84 ± 0.73[c]	2.30 ± 0.97[a]	2.33 ± 0.96[a]
Overall appearance	3.91 ± 0.81[c]	3.23 ± 0.92[ab]	3.16 ± 0.98[ab]	3.52 ± 0.85[bc]	3.06 ± 0.91[a]	3.13 ± 0.94[a]
Overall flavour	4.07 ± 0.88[c]	2.58 ± 1.05[b]	2.30 ± 1.07[ab]	3.81 ± 0.83[c]	2.13 ± 0.96[a]	2.20 ± 0.99[a]
Overall texture	3.98 ± 0.92[b]	2.83 ± 0.95[a]	2.63 ± 1.04[a]	3.58 ± 0.89[b]	2.43 ± 1.02[a]	2.65 ± 1.09[a]
Overall taste	3.99 ± 0.96[b]	2.65 ± 1.01[a]	2.41 ± 1.02[a]	3.69 ± 0.86[a]	2.24 ± 0.94[a]	2.27 ± 0.96[a]
**Texture attributes**						
Hardness	3.69 ± 0.94[c]	2.99 ± 0.96[ab]	2.87 ±1.10[ab]	3.14 ± 1.10[b]	2.65 ± 1.06[a]	2.93 ± 1.18[ab]
Crispness	3.73 ± 0.99[b]	2.88 ± 1.01[a]	2.92 ± 1.01[a]	3.01 ± 1.09[b]	2.56 ± 1.06[a]	2.76 ± 1.11[a]
Lumpiness	3.69 ± 0.90[b]	3.08 ± 1.04[a]	3.02 ± 1.01[a]	3.38 ± 0.92[ab]	2.98 ± 0.96[a]	3.10 ± 1.12[a]
Moistness	3.45 ± 1.07[b]	2.83 ± 0.91[a]	2.81 ± 0.98[a]	3.36 ± 0.94[b]	2.71 ± 0.89[a]	2.92 ± 1.05[a]
**Flavour attributes**						
Salty flavour	3.29 ± 1.05[c]	2.69 ± 1.04[a]	2.62 ± 1.12[a]	3.15 ± 0.97[bc]	2.70 ± 1.06[ab]	2.71 ± 1.07[ab]
Butter flavour	3.44 ±1.14[b]	2.79 ± 1.10[a]	2.59 ± 0.96[a]	3.27 ± 0.97[b]	2.58 ± 0.95[a]	2.52 ± 1.09[a]
Greasiness	3.34 ± 1.12[b]	2.60 ± 0.95[a]	2.44 ± 0.97[a]	3.31 ± 1.13[a]	2.49 ± 1.00[a]	2.60 ± 1.03[a]
Odour	3.44 ±1.14[b]	2.34 ± 1.01[a]	2.16 ± 1.07[a]	3.40 ± 1.14[a]	2.21 ± 1.14[a]	2.12 ±1.01[a]
Rancid Odour	3.33 ± 1.12[b]	2.58 ± 1.12[a]	2.31 ± 1.03[a]	3.33 ± 1.22[a]	2.37 ± 1.10[a]	2.28 ± 1.07[a]
**Colour attributes**						
Colour difference	3.67 ± 0.96[c]	3.18 ± 0.98[ab]	2.96 ± 1.05[a]	3.39 ± 0.90[bc]	3.08 ± 0.82[ab]	3.07 ± 0.92[ab]
Lightness difference	3.68 ± 1.00[c]	3.12 ± 0.98[ab]	2.89 ± 1.08[a]	3.41 ± 0.94[bc]	3.08 ± 0.85[ab]	3.15 ± 0.94[ab]
**Weight mean value**	**3.63**	**2.81**	**2.65**	**3.39**	**2.59**	**2.66**

Results are given as mean ± standard deviation (n=86); Values in the same row with the same letter are not statistically different ($p < 0.05$).

Supplementary Figure

Figure S1. Bacteria number of snack bars during storage

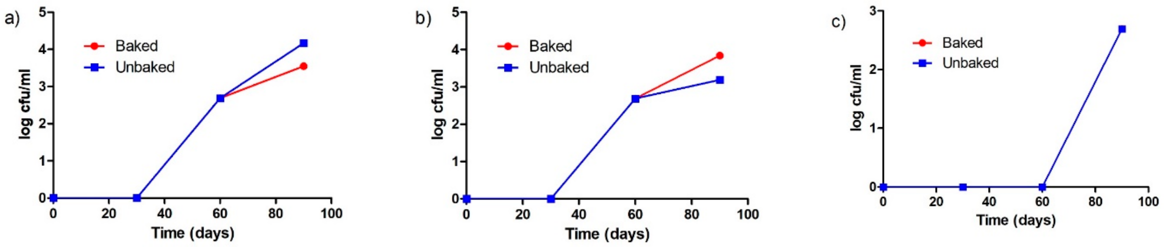

Figure S2. Mold number of snack bars during storage

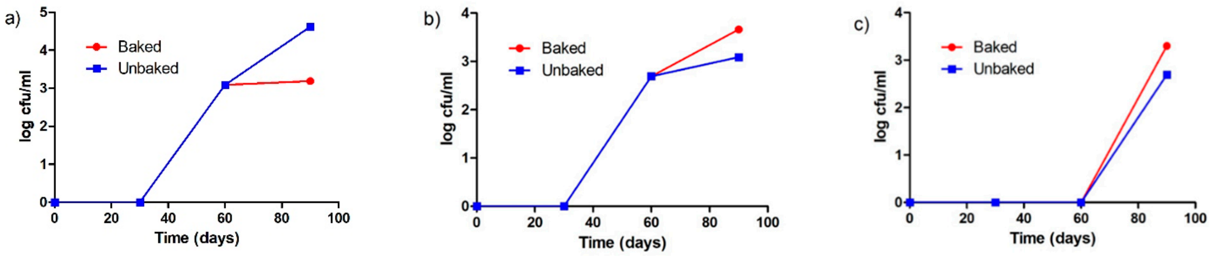

Figure S3. Water activity of snack bars during storage

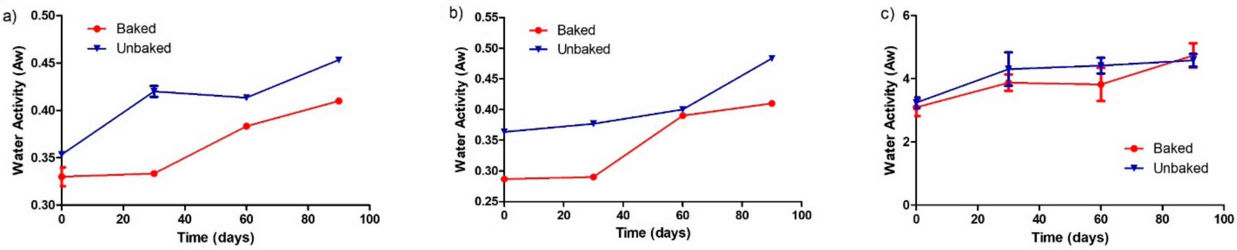

Figure S4. Moisture content of snack bars during storage

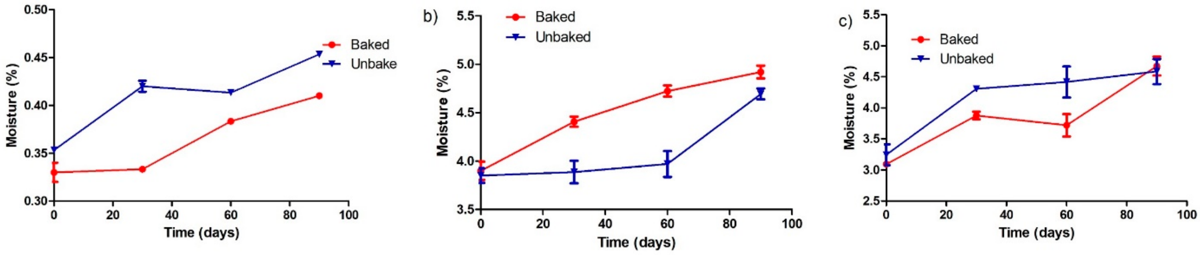

Figure S5. Color changes (ΔE) of snack bars during storage

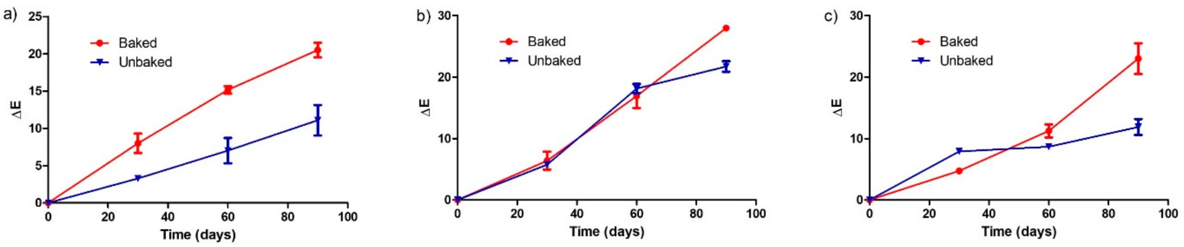

References

1. Hansen, L.; Rose, M.S. Sensory Acceptability is Inversely Related to Development of Fat Rancidity in Bread Made from Stored Flour. *J Am Diet Assoc.* **1996**, *96*, 792-793, doi:10.1016/s0002-8223(96)00218-0.
2. Jensen, S.; Skibsted, L.H.; Kidmose, U.; Thybo, A.K. Addition of cassava flours in bread-making: Sensory and textural evaluation. *LWT - Food Science and Technology* **2015**, *60*, 292-299, doi:10.1016/j.lwt.2014.08.037.
3. Kim, J.H.; Lee, H.J.; Lee, H.-S.; Lim, E.-J.; Imm, J.-Y.; Suh, H.J. Physical and sensory characteristics of fibre-enriched sponge cakes made with Opuntia humifusa. *LWT* **2012**, *47*, 478-484, doi:10.1016/j.lwt.2012.02.011.
4. Ramírez-Jiménez, A.K.; Gaytán-Martínez, M.; Morales-Sánchez, E.; Loarca-Piña, G. Functional properties and sensory value of snack bars added with common bean flour as a source of bioactive compounds. *LWT* **2018**, *89*, 674–680, doi:10.1016/j.lwt.2017.11.043.
